# A ROS-Based Online System for 3D Gaussian Splatting Optimization: Flexible Frontend Integration and Real-Time Refinement

**DOI:** 10.3390/s25134151

**Published:** 2025-07-03

**Authors:** Li’an Wang, Jian Xu, Xuan An, Yujie Ji, Yuxuan Wu, Zhaoyuan Ma

**Affiliations:** 1 School of System Design and Intelligent Manufacturing, Southern University of Science and Technology, Shenzhen 518055, China; 12232625@mail.sustech.edu.cn (L.W.);; 2 K-CLUB University College, Korea University, Seoul 14779, Republic of Korea

**Keywords:** 3D reconstruction, SLAM, 3D Gaussian splatting, bundle adjustment

## Abstract

The 3D Gaussian splatting technique demonstrates significant efficiency advantages in real-time scene reconstruction. However, when its initialization process relies on traditional SfM methods (such as COLMAP), there are obvious bottlenecks, such as high computational resource consumption, as well as the decoupling problem between camera pose optimization and map construction. This paper proposes an online 3DGS optimization system based on ROS. Through the design of a loose-coupling architecture, it realizes real-time data interaction between the frontend SfM/SLAM module and backend 3DGS optimization. Using ROS as a middleware, this system can access the keyframe poses and point-cloud data generated by any frontend algorithms (such as ORB-SLAM, COLMAP, etc.). With the help of a dynamic sliding-window strategy and a rendering-quality loss function that combines L1 and SSIM, it achieves online optimization of the 3DGS map. The experimental data shows that compared with the traditional COLMAP-3DGS process, this system reduces the initialization time by 90% and achieves an average PSNR improvement of 1.9 dB on the TUM-RGBD, Tanks and Temples, and KITTI datasets.

## 1. Introduction

Recently, 3D Gaussian Splatting (3DGS) [1] has emerged as a pivotal technique for generating dense scene representations from sparse points, offering superior efficiency for real-time applications. A critical bottleneck in 3DGS lies in its initialization, where conventional SfM approaches like COLMAP [2] impose extensive computational overheads via bundle adjustment (BA), which optimizes the 3DGS map quality without explicit consideration for the camera pose. To address this, we introduce Local Gaussian Splatting Bundle Adjustment (LGSBA), a two-stage optimization framework that first refines poses via traditional BA and then employs a sliding window with rendering-quality loss to enhance Gaussian map fidelity. Coupled with an ORB-SLAM-based online reconstruction pipeline via ROS, our system reduces the initialization time by 10× while improving 3DGS map PSNR. The key contributions include the following:

1. A Tightly Coupled ORB-SLAM and 3DGS Optimization System: We first propose a ROS-based tightly coupled framework that injects real-time local bundle adjustment (Local BA) results from ORB-SLAM into the 3DGS optimization pipeline. By dynamically acquiring keyframe poses and point cloud updates, this framework enables incremental optimization of the Gaussian map, reducing the initialization time overhead by 90% compared to traditional COLMAP workflows. Leveraging ORB-SLAM’s real-time localization capabilities, the system establishes a closed-loop between localization and mapping. This integration not only accelerates the 3DGS initialization but also ensures high-quality initial inputs for Gaussian map construction through real-time feature tracking and scene understanding.
2. Local Gaussian Splatting Bundle Adjustment (LGSBA): We propose an LGSBA optimization framework based on a sliding window, which dynamically refines local viewpoint poses by integrating a rendering-quality loss function that aggregates errors from all keyframes within the window (combining L1 loss and SSIM). This algorithm adaptively adjusts the Gaussian parameters and proposes to balance pose accuracy with map rendering quality, coupling the local structure refinement of the Gaussian map with camera pose optimization to mitigate map blurring caused by projection errors in various scenes. Experiments across three datasets—TUM-RGBD, Tanks and Temples, and KITTI—demonstrate an average PSNR improvement of 1.9 dB.
3. An Open-Source Codebase: The core algorithms, including ORB-SLAM initialization, LGSBA optimization, and the tightly coupled ROS framework, are open source and available at https://github.com/wla-98/worse-pose-but-better-3DGS, accessed on 29 June 2025, enabling the reproducibility of the proposed method and facilitating further research on 3D Gaussian splatting optimization. The repository includes implementation details for real-time initialization, sliding window-based bundle adjustment, and online map refinement, providing a comprehensive resource for the research community.

## 2. Related Work

### 2.1. 3D Gaussian Splatting

Benefiting from its explicit representation, short training time, and real-time rendering speed, 3DGS [1] has quickly outpaced Neural Radiance Fields [3] to become a prominent research focus in the field of 3D reconstruction. Numerous studies aim to enhance the pixel-level scene quality generated by 3DGS, which remains a key objective of 3DGS optimization.

Some studies [4,5] focus on optimizing the original parameters of 3DGS. However, parameter adjustments often require extensive experimentation and are closely tied to the specific characteristics of a given scene, limiting their generalizability. Other studies [6,7,8] improve rendering quality by incorporating depth estimation information, but acquiring depth data involves additional resource costs.

Despite these advancements, limited research has specifically explored how the initialization of camera poses and sparse point clouds impacts subsequent 3DGS results. Furthermore, most 3DGS reconstruction rely on the COLMAP [2] pipeline, which consumes substantial computational resources when processing large image sets, significantly reducing the efficiency of scene reconstruction. Addressing this limitation is a key objective of this article.

### 2.2. Visual SLAM

Direct SLAM methods [9,10,11,12] excel in handling low-texture and dynamic environments. However, due to their assumption of consistent grayscale intensity, these methods are highly sensitive to illumination changes and noise, which makes them less suitable for real-world applications. Feature-based SLAM [13,14,15,16,17,18] is highly robust, delivering excellent real-time performance and supporting multi-task parallel processing to construct high-precision maps. These systems integrate seamlessly with data from other sensors, providing accurate and reliable solutions for localization and mapping. However, they produce sparse point cloud maps, which hinder downstream tasks and demand considerable time for parameter tuning.

NeRF-based SLAM [3,19,20,21,22,23,24] excels in 3D dense modeling, rendering novel viewpoints, and predicting unknown regions, significantly enhancing 3D model generation and processing under optimal observational inputs. Nevertheless, these methods depend on large amounts of data and computational resources for neural network training, making real-time performance difficult to achieve. Furthermore, their implicit representations are less suited for downstream tasks.

Over the past two years, numerous studies have proposed SLAM systems based on 3DGS with explicit volumetric representations. Most of these systems [25,26,27,28,29,30,31,32] rely on 3D reconstruction frameworks, such as ORBSLAM [14,15], DROID-SLAM [33], or DUSt3R [34] to provide camera poses and initial point clouds for initializing the Gaussian map. Alternatively, they require depth information corresponding to RGB images as a prior to constrain the map reconstruction process. MonoGS [35] stands out by eliminating dependency on other SLAM systems for initialization. It unifies 3D Gaussian representations as the sole three-dimensional model while simultaneously optimizing the Gaussian map, rendering novel viewpoints, and refining camera poses.

However, all such systems face a critical limitation: achieving both higher-precision camera pose optimization and dense map reconstruction using only a monocular camera is challenging. This article clarifies the underlying reasons behind these limitations and proposes a novel approach that combines ORBSLAM initialization with LGSBA for pose optimization. This integration enhances the 3DGS framework, enabling it to achieve better results in terms of Gaussian map and rendered images across various scenarios.

## 3. Method

### 3.1. ORB-SLAM Initialization

The ORB-SLAM initialization process selects keyframes and optimizes poses via local/global BA. The core optimization is:

(1) $$\left\{T_{K_{i}}^{*},P_{j}^{*}\right\}=\underset{T_{K_{i}},P_{j}}{\arg\;\min}\sum_{j=1}^{N_{i}}\sum_{i=1}^{N_{k}}\rho (||\pi \left(T_{K_{i}}P_{j}\right)-f_{i,j}{\left|\right|}^{2}),$$

keyframe criteria and pose parameterization are detailed in Appendix A, while 3D points are extended with spherical harmonics for color representation.

### 3.2. 3DGS Fundamental Model

3DGS represents scenes using Gaussians:

(2) $$G\left(x\right)=e^{-\frac{1}{2}{\left(x-\mu \right)}^{T}\Sigma^{-1}\left(x-\mu \right)},$$

with covariance parameterized as $\Sigma =RSS^{T}R^{T}$ (Appendix B). Two-dimensional projection and α-blending rendering are described in Appendix B.

### 3.3. Local Gaussian Splatting Bundle Adjustment (LGSBA) and Mathematical Derivations

The LGSBA framework employs a sliding-window strategy to balance camera pose accuracy and Gaussian map rendering quality, rooted in the mathematical properties of the special Euclidean group SE(3). For a keyframe pose T∈SE(3) and its Lie algebra τ∈se(3), the left Jacobian is defined as:

(3) $$\frac{Df(T)}{DT}≜\lim_{\tau \to 0}\frac{\log\left(f\left(\exp\left(\tau \right)\circ T\right)\circ f{\left(T\right)}^{-1}\right)}{\tau },$$

forming the basis for gradient computation in pose optimization. Here, SE(3) represents the group of rigid body transformations, combining rotation and translation, while se(3) is its corresponding Lie algebra.

The original single-keyframe loss function is:

(4) $$L=\lambda L_{1}+\left(1-\lambda \right)\;\mathrm{SSIM},$$

where $L_{1}$ is the pixel-wise L1 loss, SSIM measures structural similarity, and λ weights their contributions. LGSBA extends this to a multi-keyframe sliding window (e.g., N=7 frames: current frame +3 before/after), with total loss:

(5) $$L_{\mathrm{total}}=\sum_{i\in W}\left(\lambda L_{1}^{\left(i\right)}+\left(1-\lambda \right)\;{\mathrm{SSIM}}^{\left(i\right)}\right),$$

where *W* denotes the keyframe index set.

For gradient optimization on SE(3), the 2D projection derivative with respect to pose $T_{K_{l}}$ is:

(6) $$\frac{\partial P_{j}^{2D}}{\partial T_{K_{l}}}=\frac{\partial P_{j}^{2D}}{\partial P_{j}}\frac{DP_{j}}{DT_{K_{l}}},$$

with the pose derivative of 3D point $P_{j}$ derived via Lie algebra:

(7) $$\frac{DP_{j}}{DT_{K_{i}}}=\left[\begin{array}{cc} I & -P_{j}^{\times } \end{array}\right].$$

Here, $P_{j}^{\times }$ denotes the skew-symmetric matrix of the 3D point $P_{j}$, and *I* is the identity matrix.

Similarly, the covariance derivative is:

(8) $$\frac{\partial \Sigma_{j}^{2D}}{\partial T_{K_{l}}}=\frac{\partial \Sigma_{j}^{2D}}{\partial J_{K_{l}}}\frac{\partial J_{K_{l}}}{\partial P_{j}}\frac{DP_{j}}{DT_{K_{l}}},$$

where the rotational component derivative is:

(9) $$\frac{DR_{K_{i}}}{DT_{K_{i}}}=\left[\begin{array}{cc} 0 & -R_{K_{i}\left(:,1\right)}^{\times }\\ 0 & -R_{K_{i}\left(:,2\right)}^{\times }\\ 0 & -R_{K_{i}\left(:,3\right)}^{\times } \end{array}\right].$$

In this context, $R_{K_{i}}$ is the rotation matrix of keyframe $K_{i}$, and $R_{K_{i}\left(:,k\right)}$ denotes the *k*-th column of $R_{K_{i}}$. The operator D represents the derivative with respect to the pose $T_{K_{i}}$, while the skew-symmetric matrix $R_{K_{i}\left(:,k\right)}^{\times }$ enables the conversion between vector and matrix representations for rotational operations.

The total loss gradient under LGSBA becomes:

(10) $$\frac{\partial L_{\mathrm{total}}}{\partial T_{K_{l}}}=\sum_{i\in W}\left(\lambda \frac{\partial L_{1}^{\left(i\right)}}{\partial T_{K_{l}}}+\left(1-\lambda \right)\frac{\partial {\mathrm{SSIM}}^{\left(i\right)}}{\partial T_{K_{l}}}\right),$$

with gradient components:

(11) $$\frac{\partial L_{1}^{\left(i\right)}}{\partial T_{K_{l}}}=\frac{\partial P_{j}^{2D(i)}}{\partial T_{K_{l}}},\;\frac{\partial {\mathrm{SSIM}}^{\left(i\right)}}{\partial T_{K_{l}}}=\frac{\partial \Sigma_{j}^{2D(i)}}{\partial T_{K_{l}}}.$$

The LGSBA workflow follows a dual stage:

1. Traditional BA Refinement: Conventional bundle adjustment optimizes poses for geometric accuracy, where $T_{K_{i}}$ represents the camera pose of keyframe $K_{i}$, and $P_{j}$ are 3D map points.

2. Sliding-Window Optimization: $L_{\mathrm{total}}$ refines poses to enhance rendering fidelity, leveraging SE(3) gradient properties to balance pose precision and map quality, as validated in Section 3.3.

### 3.4. System Workflow Based on the Diagram

**Figure 1:**
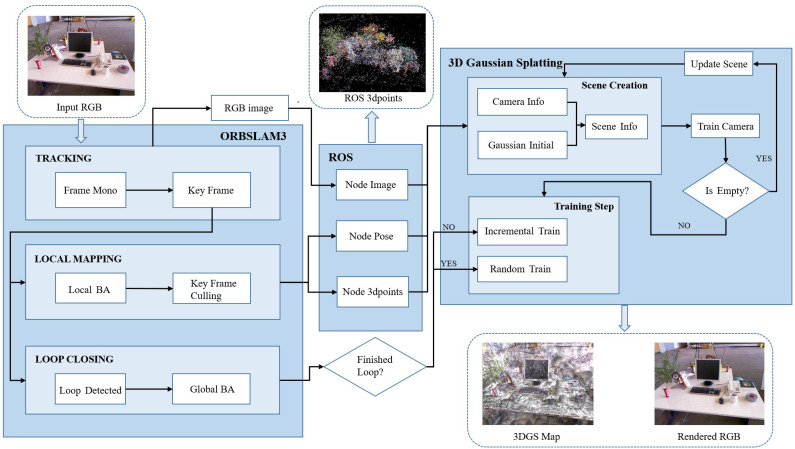
System overview: Our online optimization system achieves tight coupling between ORB-SLAM3 and the 3DGS system through ROS. By leveraging ORB-SLAM3, we optimize keyframe extraction and pose estimation, while ROS facilitates real-time data exchange for incremental 3DGS training. Global loop closure triggers comprehensive map refinement, balancing local accuracy and global consistency.

As illustrated in Figure 1, the complete system workflow is structured as follows:

#### 3.4.1. Input and Frontend Initialization (ORB-SLAM3 Leading)

The system takes RGB images as input and executes the Tracking, Local Mapping, and Loop Closing processes via ORB-SLAM3:

1. Tracking: Monocular frames (Frame Mono) filter keyframes (Key Frame), providing basic data for subsequent optimization.
2. Local Mapping: This optimizes keyframe poses through local bundle adjustment (Local BA) and refines data with keyframe culling (Key Frame Culling).
3. Loop Closing: This triggers global bundle adjustment (Global BA) upon loop detection (Loop Detected) to correct accumulated errors, outputting optimized keyframe images, poses, and sparse point clouds (Node Image, Node Pose, Node 3D points).

#### 3.4.2. Data Interaction and Middleware (ROS Bridging)

Leveraging ROS nodes, the Local Mapping thread of ORB-SLAM3 publishes real-time local BA results (keyframe images, poses, map points). After global BA completion by the Loop Closing thread, data updates are triggered, enabling tight coupling interaction between ORB-SLAM3 and the 3DGS system.

#### 3.4.3. 3DGS Scene Construction and Optimization

Upon receiving data from ROS, the 3DGS system executes sequential operations tailored to ORB-SLAM’s loop-closing status:

1. Scene Creation: This generates scene information (Scene Info) by combining camera parameters (Camera Info) and Gaussian initialization (Gaussian Initial), initializing the 3DGS environment.
2. Training Strategy Selection:
  (a) Incremental Training (No Global Loop): When ORB-SLAM has not detected a global loop (partial keyframes processed), LGSBA optimization is employed. It uses sliding-window loss ($L_{\mathrm{total}}$) to refine Gaussian parameters and camera poses via backpropagation and updates the map incrementally with new keyframes to maintain local consistency.
  (b) Random Training (Global Loop Detected): After global loop closure (all keyframes processed), the original 3DGS random optimization is selected. It uses random sampling of scene cameras for global map refinement and corrects accumulated errors to ensure global consistency.
3. Scene Update Loop: This continuously refines Gaussian map parameters during incremental training, terminates incremental training upon global loop detection, triggering random optimization, and outputs the final 3DGS map and rendered RGB images after global optimization.

#### 3.4.4. Loop Closure and Robustness Enhancement

Utilizing ORB-SLAM3’s global BA capability, the system introduces randomly selected scene cameras for training post-loop closure. This compensates for local optimization biases, enhancing both system robustness and 3DGS map reconstruction quality.

In summary, the system completes frontend pose and sparse map optimization via ORB-SLAM3, enables real-time data transfer through ROS, and executes Gaussian scene construction with LGSBA/3DGS hybrid optimization, forming a complete “front-end vision–middleware communication–back-end reconstruction” loop. The flowchart (Figure 1) intuitively presents the module interaction logic and data flow.

## 4. Dataset and Metrics

All experiments were conducted on a Windows 11 WSL2 system, equipped with an Intel Core i7-11700K CPU (Intel, Santa Clara, CA, USA ) and an NVIDIA RTX 3090 GPU (Nvidia, Santa Clara, CA, USA).

### 4.1. Datasets Description

We evaluated our approach on three publicly available datasets: TUM-RGBD, Tanks and Temples, KITTI. These datasets offer complementary advantages in terms of scene types, motion patterns, and evaluation metrics, which enabled a comprehensive assessment of the proposed method under varied conditions.

#### 4.1.1. TUM-RGBD Dataset (TUM)

The TUM-RGBD dataset is widely used for indoor SLAM research. It contains multiple sequences of both dynamic and static indoor scenes captured using a handheld RGB-D camera. Ground-truth trajectories with high precision are provided, and common evaluation metrics include Absolute Trajectory Error (ATE) and Relative Pose Error (RPE). This dataset is ideal for assessing the stability of feature tracking in the presence of dynamic disturbances and the precision of backend optimization.

#### 4.1.2. Tanks and Temples Dataset (TANKS)

The Tanks and Temples dataset is designed for large-scale outdoor 3D reconstruction tasks. It features high-resolution scenes with complex geometry and detailed textures. This dataset is frequently used to evaluate multi-view 3D reconstruction and dense scene modeling methods, allowing us to assess the capability of our 3DGS approach in modeling radiance fields at different scales.

#### 4.1.3. KITTI Dataset (KITTI)

The KITTI dataset was collected from a vehicle-mounted platform and is primarily targeted at SLAM and visual odometry research in real-world traffic scenarios. It provides continuous image sequences along with high-precision GPS/IMU data, making it suitable for evaluating the system’s ability to suppress accumulated errors over long-term operations and its robustness in challenging road environments.

The complementary nature of these datasets—in terms of indoor/outdoor settings, different motion modalities (handheld, vehicle, aerial), and diverse evaluation metrics (ATE/RPE)—enables a systematic evaluation of

1. The robustness of the SLAM frontend under dynamic disturbances;
2. The radiance field modeling capabilities of 3DGS in scenes of varying scales;
3. The suppression of cumulative errors during long-term operations.

### 4.2. Evaluation Metrics

To quantitatively analyze the reconstruction results, we adopted several evaluation metrics that measure the differences between the reconstructed images and ground truth, the accuracy of camera poses, and the geometric precision. The metrics used are as follows:

1. L1 Loss: This metric evaluates image quality by computing the pixel-wise absolute difference between the reconstructed image and the ground truth image. A lower L1 loss indicates a smaller discrepancy and, consequently, better reconstruction quality:
  (12) $$L_{1}=\frac{1}{N}\sum_{i=1}^{N}\left|I_{\mathrm{reconstructed}}\left(i\right)-I_{\mathrm{gt}}\left(i\right)\right|,$$
  where *N* is the total number of pixels.
2. PSNR: PSNR measures the signal-to-noise ratio between the reconstructed and ground truth images, expressed in decibels (dB). A higher PSNR value indicates better image quality. It is computed as:
  (13) $$\mathrm{PSNR}=10\log_{10}\left(\frac{R^{2}}{MSE}\right),$$
  with *R* being the maximum possible pixel value (typically 255 for 8-bit images) and MSE defined as:
  (14) $$MSE=\frac{1}{N}\sum_{i=1}^{N}{\left(I_{\mathrm{reconstructed}}\left(i\right)-I_{\mathrm{gt}}\left(i\right)\right)}^{2}.$$
3. SSIM: SSIM measures the structural similarity between two images by considering luminance, contrast, and texture. Its value ranges from −1 to 1, with values closer to 1 indicating higher similarity:
  (15) $$SSIM\left(x,y\right)=\frac{\left(2\mu_{x}\mu_{y}+C_{1}\right)\left(2\sigma_{xy}+C_{2}\right)}{\left(\mu_{x}^{2}+\mu_{y}^{2}+C_{1}\right)\left(\sigma_{x}^{2}+\sigma_{y}^{2}+C_{2}\right)},$$
  where $\mu_{x},\mu_{y}$ are the means, $\sigma_{x}^{2},\sigma_{y}^{2}$ the variances, and $\sigma_{xy}$ the covariance of images *x* and *y*, with $C_{1}$ and $C_{2}$ being small constants.
4. LPIPS: LPIPS assesses perceptual similarity between images using deep network features. It is computed as:
  (16) $$\mathrm{LPIPS}\left(I_{\mathrm{reconstructed}},I_{\mathrm{gt}}\right)=\frac{1}{K}\sum_{k=1}^{K}{∥\phi_{k}\left(I_{\mathrm{reconstructed}}\right)-\phi_{k}\left(I_{\mathrm{gt}}\right)∥}_{2},$$
  where $\phi_{k}\left(\;\cdot \;\right)$ represents the feature map extracted from the *k*-th layer of a deep convolutional network, and *K* is the total number of layers. A lower LPIPS indicates a smaller perceptual difference.
5. APE: APE quantifies the geometric precision of the reconstructed camera poses by computing the Euclidean distance between the translational components of the estimated and ground truth poses. For *N* poses, it is defined as:
  (17) $$APE=\frac{1}{N}\sum_{i=1}^{N}∥t_{\mathrm{reconstructed}}\left(i\right)-t_{\mathrm{gt}}\left(i\right)∥.$$
  If rotation errors are also considered, APE can be extended as:
  (18) $$APE_{\mathrm{SE}(3)}=\frac{1}{N}\sum_{i=1}^{N}\left(∥t_{i}^{\mathrm{err}}∥+\beta ∥r_{i}^{\mathrm{err}}∥\right),$$
  where $r_{i}^{\mathrm{err}}$ is the axis-angle error for rotation, and β is a weighting factor (typically set to 1).
6. RMSE: RMSE measures the average error between the reconstructed and ground truth camera poses, computed as:
  (19) $$RMSE=\sqrt{\frac{1}{N}\sum_{i=1}^{N}{∥T_{\mathrm{reconstructed}}\left(i\right)-T_{\mathrm{gt}}\left(i\right)∥}^{2}},$$
  where $T_{\mathrm{reconstructed}}\left(i\right)$ and $T_{\mathrm{gt}}\left(i\right)$ denote the reconstructed and ground truth poses for frame *i*, respectively.

These metrics provide a comprehensive evaluation of the image reconstruction quality, visual fidelity, and geometric accuracy. In our experiments, we used these metrics to quantitatively compare different methods and provide a solid basis for performance evaluation.

## 5. Experiment

The Section 5 is structured to validate the two primary innovations of this study: (1) the Local Gaussian Splatting Bundle Adjustment algorithm, and (2) the ROS-based tightly coupled ORB-SLAM and 3DGS system, which integrates real-time initialization with dynamic optimization. Each experiment directly demonstrates how these innovations enhance 3DGS reconstruction quality and efficiency.

### 5.1. Experiment 1: LGSBA Effectiveness Verification via 3DGS Training Comparison

This experiment aimed to evaluate the LGSBA algorithm by comparing three training strategies under the ORB-SLAM initialization framework: original 3DGS training, Scaffold-GS, and our LGSBA-enhanced approach. The core objective was to demonstrate that LGSBA—when initialized by ORB-SLAM—optimizes 3DGS rendering quality by dynamically balancing ORB-SLAM’s pre-trained camera poses (refined via its built-in traditional BA) and Gaussian map fidelity, even when introducing controlled pose errors. This directly validates the innovation of LGSBA’s two-stage optimization framework:

1. Leveraging ORB-SLAM’s Native BA: Initial refinement of camera poses using ORB-SLAM’s traditional bundle adjustment.
2. Sliding-Window Joint Optimization: Subsequent refinement of both poses and Gaussian parameters using rendering-quality loss (L1+SSIM), prioritizing map fidelity while maintaining acceptable pose accuracy.

The experimental design focused solely on the ORB-SLAM initialization paradigm. The results were expected to show that LGSBA improves rendering quality in real-time scenarios, highlighting its superiority in integrating pose optimization with 3DGS rendering quality under the ORB-SLAM framework.

#### 5.1.1. System Workflow of 3DGS Reconstruction

**Figure 2:**
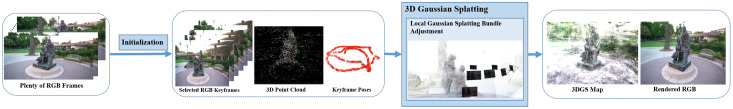
Experimental workflow for validating LGSBA’s effectiveness: integrating ORB-SLAM initialization, sliding-window optimization, and quantitative evaluation to demonstrate the tight coupling of pose accuracy and 3DGS rendering quality.

As shown in Figure 2, the workflow was structured to explicitly validate the tight coupling of ORB-SLAM and LGSBA, a key innovation of the proposed system:

1. ORB-SLAM Preprocessing: Input RGB sequences are processed to select keyframes based on tracking quality and scene dynamics, leveraging ORB-SLAM’s real-time localization capabilities. This step outputs optimized camera poses $\left\{T_{K_{i}}^{*}\right\}$ and 3D point clouds $\left\{P_{j}^{exp}\right\}$, demonstrating the efficiency of ORB-SLAM initialization compared to COLMAP.
2. Gaussian Map Initialization: Three-dimensional points are converted to Gaussian parameters:
  (20) G(μ,c,α,R,S)
  where μ is the spatial coordinate, *c* is the spherical harmonic coefficient for color encoding, and α is the opacity. This step forms the basis for 3DGS scene representation, highlighting the innovation of using ORB-SLAM’s output for rapid initialization.
3. LGSBA Sliding-Window Optimization: A seven-frame sliding window is employed for joint optimization, integrating the rendering-quality loss function:
  (21) $$L_{\mathrm{total}}=\sum_{i\in W}\left(\lambda L_{1}^{\left(i\right)}+\left(1-\lambda \right){\mathrm{SSIM}}^{\left(i\right)}\right)$$
  Here, λ balances the contributions of pixel-wise L1 loss and structural similarity (SSIM), directly reflecting the innovation of LGSBA in coupling pose optimization with rendering quality. The sliding-window strategy dynamically refines local viewpoints, mitigating map blurring caused by projection errors.
4. Quantitative Evaluation: Rendered images are compared against ground truth using metrics (PSNR, SSIM, LPIPS, APE, RMSE) to quantify the improvement in 3DGS reconstruction quality. This evaluation directly measures the impact of LGSBA’s optimization on both visual fidelity and geometric accuracy, aligning with the study’s core innovation of enhancing 3DGS rendering through pose-map co-optimization.

#### 5.1.2. Quantitative Analysis

As shown in Table 1, under 30K iterations, LGSBA achieves significant improvements: +1.9 dB average PSNR, −0.012 L1 loss, +0.068 SSIM, and −0.081 LPIPS compared to the original 3DGS with ORB-SLAM initialization. Notable gains include a 5.05 dB PSNR improvement in the TANKS-Family scene, 2.11 dB in TANKS-Train, and 7.65 dB in TANKS-Caterpillar, highlighting its effectiveness in outdoor environments.

#### 5.1.3. Qualitative Analysis

**Figure 3:**
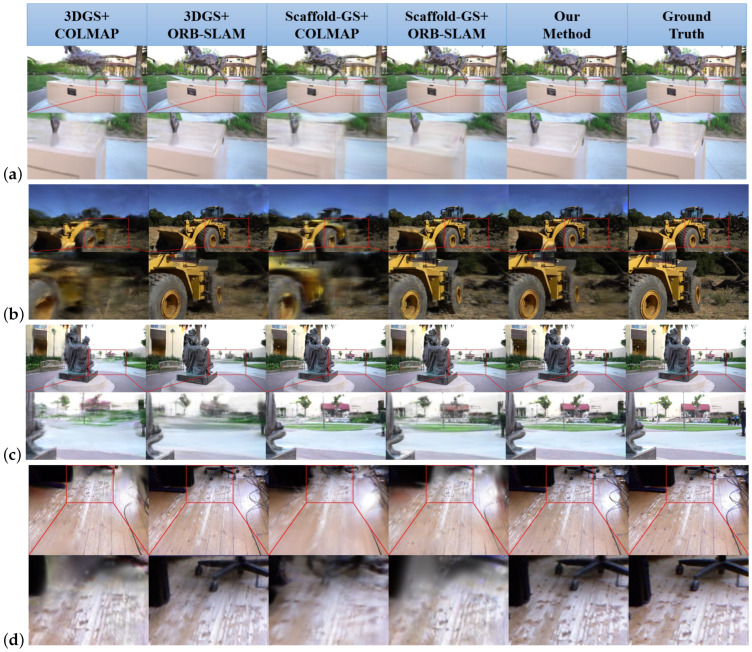
Qualitative comparison of 3DGS reconstructions: Each row pair presents full-scene rendering (top) and zoomed details (bottom) for the following: (**a**) Tank-horse, (**b**) Tank-Caterpillar, (**c**) Tank-Family, and (**d**) TUM-fg1-floor datasets. Columns represent different methods (left to right): 1. 3DGS+COLMAP; 2. 3DGS+ORB-SLAM; 3. Scaffold-GS+COLMAP; 4. Scaffold-GS+ORB-SLAM; 5. Our LGSBA method; 6. ground truth. Red boxes highlight regions where LGSBA demonstrated superior detail preservation, artifact reduction, and structural fidelity compared to baselines.

Figure 3 presents a comprehensive comparison of rendering quality across different methods and scenes. Our LGSBA-enhanced approach consistently demonstrated superior reconstruction fidelity across all datasets, particularly excelling in preserving fine-grained details and minimizing artifacts in complex scenarios. The key observations include the following:

1. Detail Preservation: LGSBA maintains sharper object contours (e.g., machinery edges in Tank-Caterpillar) and consistent surface textures (e.g., ground patterns in TUM-fg1-floor) compared to baseline methods.
2. Artifact Reduction: Noticeable reduction in rendering artifacts (blurring, floating points) in occluded regions, especially visible in Tank-Family scenes.
3. Color Consistency: More accurate color transitions and lighting reproduction, particularly evident in the Tank-horse model’s metallic surfaces.
4. Geometric Integrity: Improved depth perception and structural coherence in zoomed segments (red boxes), validating LGSBA’s effectiveness in local map refinement.

#### 5.1.4. Camera Pose Evaluation

This section evaluates LGSBA’s impact on pose estimation and its relationship with 3DGS rendering quality. The quantitative analysis of pose errors (Table 2) revealed a consistent trade-off: while LGSBA optimization increased Absolute Pose Error (APE) and Root Mean Square Error (RMSE) in most scenarios, e.g., APE in TUM-fg2-desk rises from 0.46 cm to 4.17 cm, it simultaneously boosted rendering quality, with Peak Signal-to-Noise Ratio (PSNR) increasing by up to 7.66 dB in TANKS-Caterpillar. This inverse relationship challenges conventional assumptions that a higher pose accuracy guarantees a better reconstruction quality.

**Figure 4:**
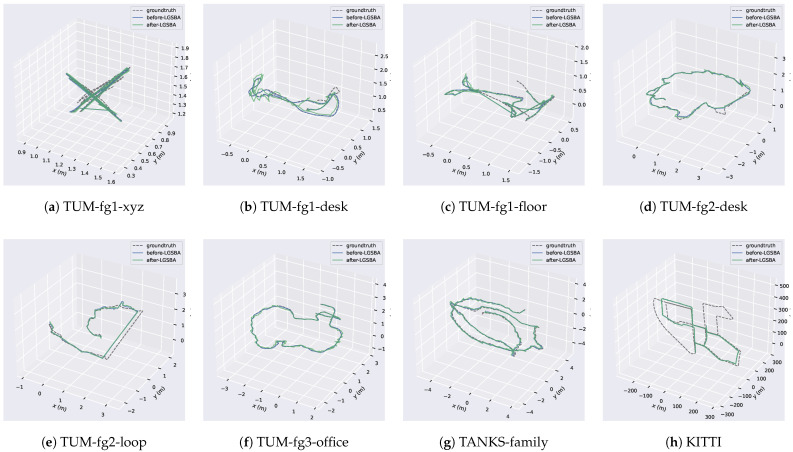
Trajectory comparison: Ground truth (black), ORB-SLAM initial poses (blue), LGSBA-optimized poses (green). GT sources: (**a**–**f**) Motion capture (TUM), (**g**) COLMAP (TANKS), (**h**) GPS-RTK (KITTI). While LGSBA increased trajectory deviation in some scenes, it improved local consistency and enabled higher-quality Gaussian maps.

Trajectory visualizations (Figure 4) provide spatial context: LGSBA-optimized trajectories show increased deviation from ground truth in structured environments like TUM-fg1-xyz. Crucially, these minor pose sacrifices yielded significant visual gains—average PSNR improved by 1.9 dB—making LGSBA particularly valuable for applications prioritizing visual fidelity over metric precision, including immersive VR/AR, dynamic scene modeling, and real-time robotic perception.

### 5.2. Online Integration of ORB-SLAM with 3DGS Map Optimization by ROS System

The proposed system establishes a tightly coupled pipeline through ROS middleware, enabling real-time data exchange between ORB-SLAM3 and 3DGS optimization modules. As illustrated in Figure 1 and specifically in Section 3.4, this integration comprises three core components:

#### 5.2.1. ORB-SLAM3 Frontend Processing

The frontend handles real-time visual odometry and mapping:

1. Tracking: Monocular frames are processed for feature extraction and keyframe selection based on scene dynamics and tracking quality.
2. Local Mapping: This performs local bundle adjustment (Local BA) to optimize keyframe poses and applies keyframe culling to remove redundancies.
3. Loop Closing: This detects loop closures to trigger global bundle adjustment (Global BA), correcting accumulated drift in poses and 3D points.

This pipeline outputs optimized keyframe images $\left\{I_{K_{i}}\right\}$, camera poses $\left\{T_{K_{i}}^{*}\right\}$, and sparse 3D point clouds $\left\{P_{j}\right\}$ at a 10 times faster initialization than offline COLMAP-based systems.

#### 5.2.2. ROS Middleware Bridging

Real-time data exchange is implemented through ROS topics/services:

1. The Local Mapping thread publishes incremental updates (keyframe poses/images/map points).
2. The Loop Closing thread triggers synchronization events upon global BA completion.
3. Custom ROS nodes ensure asynchronous communication without blocking SLAM processes.

This design maintains ORB-SLAM3’s real-time performance while providing fresh inputs for 3DGS optimization.

#### 5.2.3. 3DGS Optimization with Dynamic Strategy Switching

The backend dynamically adapts training strategies based on loop closure status:

##### Incremental Training

1. Activates when no global loop is detected (partial keyframes processed);
2. Employs LGSBA with seven-frame sliding-window optimization;
3. Minimizes rendering-quality loss $L_{\mathrm{total}}$ Equation (5) via backpropagation;
4. Jointly optimizes Gaussian parameters $\Theta_{G}$ and poses $T_{K_{j}}$:
  (22) $$\left(\Theta_{G}^{*},\left\{T_{K_{j}}^{*}\right\}\right)=\underset{\Theta_{G},\left\{T_{K_{j}}\right\}}{\arg\;\min}\sum_{i\in W}\left(\lambda L_{1}^{\left(i\right)}+\left(1-\lambda \right){\mathrm{SSIM}}^{\left(i\right)}\right)$$

##### Random Training

1. Activates after global loop closure (all keyframes processed);
2. Switches to original 3DGS random optimization;
3. Uses scene-wide camera sampling for global error correction;
4. Ensures consistency across large-scale environments.

#### 5.2.4. System Advantages

This integrated framework provides the following:

1. Real-Time Capability: 10× faster initialization than COLMAP-3DGS systems.
2. Adaptive Optimization: Balances local consistency (LGSBA) and global accuracy (random training).
3. Robustness: Loop closure triggers map-wide error correction.
4. Online Performance: It achieves >25 FPS end-to-end throughput on NVIDIA RTX 3090 GPU with
  (a) ORB-SLAM frontend: 30+ FPS (CPU processing);
  (b) 3DGS optimization: 25 FPS (GPU rendering).

#### 5.2.5. Experimental Validation of System Feasibility

**Figure 5:**
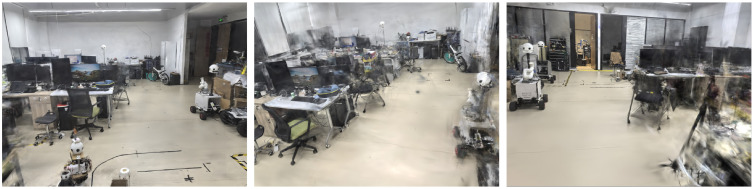
Rendered images obtained from the Gaussian map, shown from six different viewpoints.

**Figure 6:**
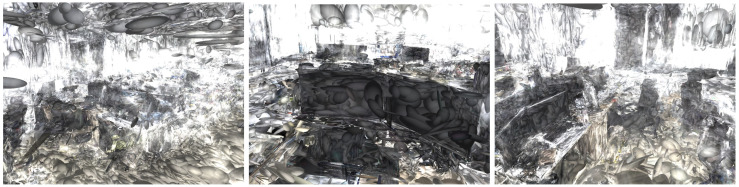
Gaussian maps observed from different viewpoints.

The laboratory scene experiments demonstrated the superiority of our online integrated system over traditional offline reconstruction methods. The quantitative results in Table 3 and qualitative visualizations in Figure 5 and Figure 6 collectively validate the system’s feasibility and advantages.

##### Quantitative Analysis

The comparison in Table 3 reveals consistent improvements across key metrics:

1. At 7K Iterations: Our online method achieved a +0.27 dB PSNR gain (+1.3%), a +0.003 SSIM improvement, and a -0.030 LPIPS reduction compared to offline reconstruction, while maintaining a comparable L1 loss.
2. At 30K Iterations: Significant quality improvements emerged:
  (a) A +0.50 dB PSNR gain (+2.1%);
  (b) A +0.012 SSIM improvement;
  (c) A −0.042 LPIPS reduction (15.2% lower).
3. Training Efficiency: The online system reached near-optimal quality (30K-level metrics) at just 7K iterations, demonstrating accelerated convergence.

##### Qualitative Analysis

**Figure 7:**
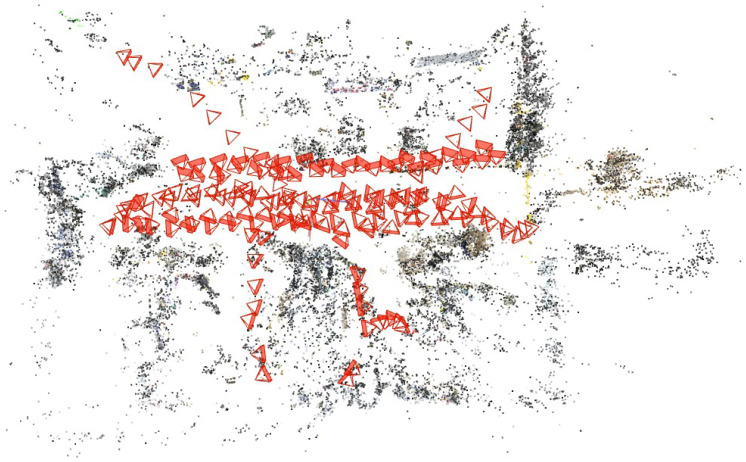
Lab point cloud and camera poses generated by ORB-SLAM for 3DGS optimization. Red triangles represent estimated camera positions, while different colored dots indicate sparse 3D points with semantic variations.

Visual evidence further confirmed the system’s effectiveness:

1. Rendering Fidelity: Figure 5 confirms
  (a) Photorealistic novel view synthesis;
  (b) Accurate lighting and material reproduction;
  (c) Clear structural details in close-up views.
2. Gaussian Map Quality: Figure 6 demonstrates
  (a) Precise geometric reconstruction of laboratory equipment;
  (b) Detailed surface representation (e.g., texture on cylindrical objects);
  (c) Minimal floating artifacts in complex areas.
3. Geometric Consistency: Figure 7 shows accurate camera pose estimation and sparse point cloud generation by ORB-SLAM, providing reliable initialization for 3DGS.

##### Conclusion on System Feasibility

The experimental results validate three critical aspects of our integrated system:

1. Real-Time Capability: It achieved a >25 FPS throughput while maintaining reconstruction quality.
2. Quality Superiority: It outperformed offline methods in perceptual metrics (PSNR/SSIM/LPIPS).
3. Operational Robustness: Consistent performance in practical indoor environments.

These findings demonstrate that our ROS-based integration of ORB-SLAM with 3DGS optimization successfully bridges the gap between real-time SLAM and high-quality neural rendering, establishing a feasible solution for 3D reconstruction applications.

## 6. Conclusions

This study proposes 3D Gaussian splatting optimization based on Local Gaussian Splatting Bundle Adjustment, a two-stage optimization framework that bridges ORB-SLAM and 3DGS via ROS middleware. The core contributions are as follows:

1. Tightly Coupled System: A ROS-based pipeline integrating ORB-SLAM’s real-time Local Bundle Adjustment with 3DGS optimization. This reduces initialization time by 90% versus COLMAP while improving average PSNR by 1.9 dB across the TUM-RGBD, Tanks and Temples, and KITTI datasets.
2. LGSBA Algorithm: A sliding-window strategy jointly optimizes rendering-quality loss ($L_{1}+\mathrm{SSIM}$) and camera poses. This balances geometric accuracy with perceptual fidelity, mitigating blurring artifacts induced by projection errors and enhancing detail preservation in complex scenes.
3. Open-Source Implementation: The released codebase (https://github.com/wla-98/worse-pose-but-better-3DGS, accessed on 29 June 2025) supports reproducibility, providing tools for real-time initialization and online map refinement.

The experiments validated LGSBA’s superiority in visual quality (e.g., +5.05 dB PSNR in TANKS-Family) and operational efficiency, establishing a practical solution for 3D reconstruction across diverse scenarios.

## 7. Limitations

Despite advancements, critical limitations remain:

1. Dynamic Environments: Reliance on ORB-SLAM’s feature tracking causes performance degradation with moving objects (e.g., pedestrians in TUM-RGBD). Dynamic elements introduce tracking errors, leading to inconsistent Gaussian map updates and rendering artifacts.
2. Illumination Sensitivity: ORB-SLAM’s susceptibility to lighting variations (e.g., outdoor shadows or flickering indoor lights) reduces pose estimation accuracy.
3. Insufficient Failure Analysis: While evaluated on diverse datasets, systematic examination of edge cases (e.g., low-texture scenes or extreme lighting) is absent. Without quantitative fault diagnosis, robustness boundaries remain unquantified.
4. Computational Overhead: Despite faster initialization, optimizing high-dimensional Gaussian parameters (covariance matrices, spherical harmonics) in ultra-large scenes imposes significant GPU memory demands, constraining real-time performance.

Future work should address these limitations by integrating dynamic object segmentation, developing illumination-invariant features, and conducting rigorous failure mode analyses.

## Figures and Tables

**Table 1:** Comparative evaluation of 3DGS reconstruction quality using three methods: (1) 3DGS with ORB-SLAM initialization, (2) Scaffold-GS [36] with ORB-SLAM initialization, and (3) our proposed method. The table reports quantitative results on various challenging scenes from TUM-RGBD, Tanks and Temples, and KITTI datasets at two training iteration counts (7K and 30K). Metrics include L1 pixel error (↓), PSNR (↑), SSIM (↑), and LPIPS (↓), where ↑ indicates larger values are better and ↓ indicates smaller values are better. Best results per metric–scenario combination are highlighted in deep red. This comprehensive evaluation demonstrates that our method consistently outperforms the other approaches, achieving superior perceptual quality (indicated by higher PSNR and SSIM, and lower L1 and LPIPS across a diverse set of scenes, specifically as illustrated in Section 5.1.

Scenes	Iterations	3DGS w. ORB-SLAM					Scaffold-GS [36] w. ORB-SLAM					LGSBA w. ORB-SLAM			
		L1 ↓	PSNR ↑	SSIM ↑	LPIPS ↓		L1 ↓	PSNR ↑	SSIM ↑	LPIPS ↓		L1 ↓	PSNR ↑	SSIM ↑	LPIPS ↓
TUM-fg2	7000	0.04445	22.7231	0.7398	0.3392		0.05892	19.5031	0.6562	0.3605		0.04147	23.2687	0.7564	0.3190
desk	30,000	0.03106	25.4048	0.8079	0.2526		0.03963	24.1913	0.7816	0.2749		0.02675	26.3698	0.8215	0.2366
TUM-fg3	7000	0.02967	25.9192	0.8702	0.2132		0.03046	26.1320	0.8495	0.2171		0.02788	26.6086	0.8754	0.1790
long office	30,000	0.02271	27.8335	0.8962	0.1739		0.02254	28.3261	0.8871	0.1710		0.02148	28.6190	0.9029	0.1448
TUM-fg2	7000	0.06844	18.6592	0.7093	0.3832		0.04203	22.7576	0.8250	0.3370		0.06613	18.4161	0.7306	0.3476
large loop	30,000	0.03502	23.5877	0.8487	0.2321		0.02675	26.5550	0.8923	0.2297		0.03297	23.9026	0.8613	0.2121
TUM-fg1	7000	0.04084	24.1946	0.7139	0.4045		0.11536	16.4651	0.5028	0.5832		0.03785	24.7616	0.7615	0.2751
floor	30,000	0.02246	28.6884	0.8123	0.2447		0.08608	18.8924	0.5747	0.4874		0.02111	29.2896	0.8756	0.1493
TUM-fg1	7000	0.04271	21.7174	0.8032	0.3109		0.02675	26.2993	0.8503	0.2593		0.03837	22.9983	0.8162	0.2823
desk	30,000	0.02467	26.6830	0.8783	0.2234		0.01646	30.6417	0.9160	0.1568		0.02117	28.1143	0.8954	0.1827
TANKS	7000	0.07196	18.4721	0.6110	0.4194		0.06984	18.6930	0.5614	0.5110		0.03922	23.3886	0.7625	0.2996
family	30,000	0.05907	19.9479	0.6985	0.3254		0.05224	20.6179	0.6758	0.3822		0.02978	24.9964	0.8308	0.2174
TANKS	7000	0.09600	16.6443	0.4398	0.6024		0.07732	18.4520	0.4906	0.5410		0.04741	22.7639	0.5953	0.4220
Caterpillar	30,000	0.07899	17.8833	0.4994	0.5322		0.06325	19.8104	0.5540	0.4780		0.02791	25.5355	0.7991	0.2577
TANKS	7000	0.04580	21.1421	0.7017	0.4393		0.05177	20.7905	0.6669	0.4058		0.03578	22.2298	0.7901	0.3279
M60	30,000	0.03388	23.1617	0.7808	0.3381		0.03472	23.8326	0.7897	0.2907		0.02820	23.7872	0.8426	0.2699
TANKS	7000	0.06553	19.7737	0.6387	0.4786		0.05711	20.6153	0.6727	0.4603		0.05074	21.1269	0.6978	0.3882
Panther	30,000	0.05116	21.5959	0.7051	0.3954		0.03952	23.4914	0.7759	0.3586		0.04139	22.7304	0.7591	0.3242
TANKS	7000	0.03704	22.3863	0.7689	0.2957		0.05187	20.8398	0.7611	0.3327		0.02885	25.0431	0.8344	0.2404
Horse	30,000	0.029178	24.2530	0.8263	0.2284		0.03315	24.6180	0.8305	0.2537		0.02376	26.4398	0.8703	0.1921
TANKS	7000	0.07377	18.7738	0.5714	0.4680		0.06106	19.5181	0.6711	0.3860		0.04691	21.4430	0.7350	0.2839
Train	30,000	0.05424	20.9735	0.6781	0.3498		0.05012	20.9398	0.7425	0.3110		0.03743	23.0867	0.8009	0.2146
TANKS	7000	0.04761	20.8189	0.7084	0.3546		0.03904	22.5495	0.7917	0.3203		0.03289	22.9611	0.8092	0.2290
Lighthouse	30,000	0.03683	22.7946	0.7728	0.2814		0.02433	26.3856	0.8633	0.2332		0.02426	25.0581	0.8507	0.1863
KITTI	7000	0.14224	13.3166	0.5003	0.5895		0.13359	14.1273	0.4716	0.6247		0.14586	13.2683	0.4808	0.6128
00	30,000	0.07117	18.0943	0.5973	0.5340		0.08427	17.3860	0.5659	0.5334		0.06340	18.5983	0.6029	0.5117
Average		0.05217	21.7478	0.7146	0.3619		0.05339	22.0166	0.7144	0.3654		0.03996	23.6464	0.7830	0.2810

**Table 2:** Quantitative evaluation of pose estimation and rendering quality across different datasets. The table presents APE and RMSE for pose estimation, along with PSNR for rendering quality. Results are reported for multiple datasets, including TUM-fg2, TUM-fg1, KITTI-00, and TANKS (Family, Caterpillar). Lower APE and RMSE values indicate more accurate pose estimation, while higher PSNR values reflect better rendering quality. The comparison highlights the effectiveness of different methods in handling diverse scenes with varying levels of complexity and motion.

Pose Estimation	Metric	TUM-fg2				TUM-fg1				Others		
		Desk	Long Office	Large w. loop		xyz	Floor	Desk		KITTI-00	TANKS-Family	TANKS-Caterpillar
Pose Estimation	APE (cm) ↓	0.4590	0.8744	7.0576		0.6736	9.2631	1.3382		14.3767	13.0878	0.1005
	RMSE (cm) ↓	0.4962	0.9844	8.5865		0.7624	10.8200	1.5237		17.4024	15.5307	0.1074
	APE (cm) ↓	4.1687	8.0663	7.7640		1.0193	3.4214	2.1502		14.4333	17.5868	0.1003
	RMSE (cm) ↓	4.9558	9.0545	8.9127		1.0869	4.1349	2.9608		17.4314	20.6191	0.1070
Quality	PSNR ↑	25.4048	27.8335	23.5877		28.1725	28.6884	26.6830		18.0943	19.9479	17.8833
	PSNR ↑	26.3698	28.6190	23.9026		28.5573	29.2896	28.1143		18.5983	24.9964	25.5355

↓ indicates lower is better, ↑ indicates higher is better.

**Table 3:** Comparative evaluation of offline reconstruction (ORB-SLAM initialization with 3DGS optimization) versus our improved online reconstruction method in a laboratory indoor scene.

Scene	Iterations	Offline Reconstruction					Online Reconstruction (Ours)			
		L1↓	PSNR↑	SSIM↑	LPIPS↓		L1↓	PSNR↑	SSIM↑	LPIPS↓
Lab	7K	0.0575	21.20	0.775	0.363		0.0467	21.47	0.778	0.333
	30K	0.0297	24.15	0.858	0.276		0.0299	24.65	0.870	0.234

↓ indicates lower is better, ↑ indicates higher is better.

## Data Availability

Data is contained within the article.

## Abbreviations

The following abbreviations are used in this manuscript:

3DGS	Three-Dimensional Gaussian Splatting
BA	Bundle Adjustment
LGSBA	Local Gaussian Splatting Bundle Adjustment
ROS	Robot Operation System
NeRF	Neural Radiance Fields
SfM	Structure-from-Motion
SOTA	State-of-the-Art
PSNR	Peak Signal-to-Noise Ratio
SSIM	Structural Similarity Index Measure

LPIPS	Learned Perceptual Image Patch Similarity
APE	Absolute Pose Error
RMSE	Root Mean Square Error
GT	Ground Truth

## Appendix A. ORBSLAM Initialization

The ORBSLAM initialization process selects a series of keyframes $\left\{K_{i}|i=1,\ldots ,N_{p}\right\}$ from a given image sequence $\left\{I_{i}|i\in N^{+}\right\}$. The selection criteria for each keyframe $K_{i}$ are based on the system’s current tracking quality, the degree of scene change, and the distribution and update of map points. Camera poses and map points are optimized through extensive local bundle adjustment and global bundle adjustment operations. This process can be mathematically expressed and simplified as follows:

(A1) $$\left\{T_{K_{i}}^{*},P_{j}^{*}\right\}=\underset{T_{K_{i}},P_{j}}{\arg\;\min}\sum_{j=1}^{N_{i}}\sum_{i=1}^{N_{k}}\rho (||\pi \left(T_{K_{i}}P_{j}\right)-f_{i,j}{\left|\right|}^{2}),$$

where $N_{i}$ denotes the number of feature points used for optimization within the keyframe $K_{i}$, $N_{k}$ represents the number of keyframes in the current window (may including all images), ρ is the robust Huber cost function, employed to mitigate the influence of outliers, π(·) refers to the projection operation that maps points from 3D space to the 2D image plane, $f_{i,j}$ is the observed pixel coordinate of map point $P_{j}=\left(x_{j},y_{j},z_{j}\right)$ in keyframe $K_{i}$, and the camera pose $T_{K_{i}}$ is represented in the following matrix form:

(A2) $$T_{k_{i}}=\left[\begin{array}{cc} R_{k_{i}} & t_{k_{i}}\\ 0 & 1 \end{array}\right],$$

where $R_{k_{i}}\in SO\left(3\right).$ is the rotation matrix, and $t_{k_{i}}\in R^{3}$ is the translation vector.

We further extend the 3D map points $P_{j}$ to $P_{j}^{exp}=\left(x_{j},y_{j},z_{j},r_{j},g_{j},b_{j}\right)\in R^{6}$ by incorporating the RGB information from the image, aligning the 3D map points with their corresponding 2D image points. Along with all optimized keyframe pose information $\left\{T_{i}^{*}|i=1,\ldots ,N_{p}\right\}$ and the RGB images associated with the keyframes, this information is jointly output.

## Appendix B. 3DGS Fundamental Model

3DGS provides an explicit and compact representation of 3D structures. Each Gaussian retains the spatial coordinates μ=(x,y,z) derived from the initialization point cloud and a covariance matrix Σ, defined as:

(A3) $$G\left(x\right)=e^{-\frac{1}{2}{\left(x-\mu \right)}^{T}\Sigma^{-1}\left(x-\mu \right)},$$

while encoding color information through spherical harmonics (SH) coefficients c obtained from the image’s RGB data. In addition, opacity α is also encoded. To ensure the positive definiteness of Σ, it is parameterized using a rotation matrix *R* and a scaling matrix *S*, i.e.,

(A4) $$\Sigma =R\;S\;S^{T}\;R^{T}.$$

Thus, a single 3D Gaussian is defined as G(μ,c,α,R,S), where μ represents the 3D mean position, c the SH color coefficients, α the opacity, *R* the rotation matrix, and *S* the scaling matrix.

After system initialization, given the set of all 3D points $\left\{P_{K_{i}}|i=1,\ldots ,N_{p}\right\}$ observable by a keyframe $K_{i}$ with pose $T_{K_{i}}^{*}$, the set of 3D Gaussians initialized from keyframe $K_{i}$ is defined as:

(A5) $$G_{K_{i}}=\left\{G\left(P_{j},c_{j},\alpha_{j},R_{j},S_{j}\right)|j=1,\ldots ,N_{i}\right\}.$$

During the 3DGS training process, a subset of these Gaussians is randomly selected for optimization. Using the keyframe poses obtained from the ORB-SLAM initialization, all 3D Gaussians observable from the selected keyframe $K_{i}$ are projected onto the camera’s 2D plane. The projected 2D position $P_{j}^{2D}$ of a 3D Gaussian $P_{j}$ is computed using a pose transformation $T_{K_{i}}$. However, calculating the projected covariance $\Sigma_{j}^{2D}$ requires an affine approximation of the projective transformation [37], expressed as:

(A6) $$\Sigma_{j}^{2D}=J_{K_{i}}R_{K_{i}}\Sigma R_{K_{i}}^{T}J_{K_{i}}^{T},$$

where $R_{K_{i}}$ denotes the rotational component of the keyframe pose $T_{K_{i}}^{*}$ and $J_{K_{i}}$ is the Jacobian corresponding to the affine approximation. This substitution, however, introduces errors—errors that are especially pronounced and uneven in outdoor scenes. Moreover, highly accurate physical-world poses can sometimes result in a poorer Gaussian map, thereby degrading the optimization quality.

The projected position $P_{j}^{2D}$ and covariance $\Sigma_{j}^{2D}$ of each 3D Gaussian determine its contribution to the rendered image. For any pixel $C_{K_{i}}^{k}$ on the keyframe image plane, all 3D Gaussians that are projected onto that pixel are sorted based on their distance from the camera and indexed as 1,…,M. These Gaussians form a set:

(A7) $$\{G_{K_{i}}^{m}\left(P_{j},c_{j},\alpha_{j},R_{j},S_{j}\right)|1\leq m\leq M\}.$$

Using the color and opacity information of these Gaussians, the final pixel color $C_{K_{i}}^{k}$ is computed via α-blending:

(A8) $$C_{K_{i}}^{k}=\sum_{m=1}^{M}c_{K_{i}}^{m}\alpha_{K_{i}}^{m}\prod_{n=1}^{m-1}\left(1-\alpha_{K_{i}}^{n}\right),$$

which is performed for every pixel to render the image $I_{K_{i}}^{pred}$ from the keyframe’s perspective. Subsequently, a loss function is used to measure the difference between the rendered image $I_{K_{i}}^{pred}$ and the ground truth image $I_{K_{i}}^{true}$.

## Appendix C. Error Decomposition and Covariance Propagation Analysis

During the projection process, the transformed covariance matrix $\Sigma_{j}^{2D}$ of each 3D Gaussian is influenced by both camera pose perturbations and intrinsic Gaussian parameter variations. Let the pose perturbation be represented as δT∈SE(3), with its Lie algebra representation $\xi ={\left[\phi^{T},\rho^{T}\right]}^{T}$, where ϕ and ρ are the rotational and translational perturbations, respectively. The Jacobian matrix $J_{K_{i}}$ of the affine projection can then be differentiated with respect to ξ as:

(A9) $$\frac{\partial J_{K_{i}}}{\partial \xi }=\left[\frac{\partial J_{K_{i}}}{\partial \phi },\frac{\partial J_{K_{i}}}{\partial \rho }\right],$$

where $\frac{\partial J_{K_{i}}}{\partial \phi }$ reflects the nonlinear impact of rotational changes, and $\frac{\partial J_{K_{i}}}{\partial \rho }$ corresponds to the linear sensitivity to translations.

When the pose changes from $T_{K_{i}}^{*}$ to $T_{K_{i}}^{*}\exp\left(\xi \right)$, the projected 2D covariance variation can be approximated by:

(A10) $$\Delta \Sigma_{j}^{2D}\approx \frac{\partial \Sigma_{j}^{2D}}{\partial \xi }\delta \xi +\frac{\partial \Sigma_{j}^{2D}}{\partial \Sigma }\Delta \Sigma ,$$

where the first term captures the propagated error due to pose perturbation, and the second term accounts for internal Gaussian parameter changes (e.g., scaling matrix *S*, rotation matrix *R*). This decomposition explicitly shows that the total rendering error consists of two interacting components—pose-induced and Gaussian-induced—coupled via the Jacobian $J_{K_{i}}$.

## Appendix D. Nonlinear Pose-Map Interplay

The optimization of 3DGS parameters under pose uncertainty demonstrates a nonlinear interplay. In theory, accurate poses minimize projection errors. However, in practice, slight pose deviations can enhance rendering quality via two mechanisms:

1. Covariance Alignment Across Views: Pose adjustments may lead to better alignment of projected Gaussians across multiple keyframes, reducing blurring effects during multi-view fusion.
2. SSIM Reweighting: Perturbed poses can improve the structural similarity (SSIM) between projected Gaussians and ground-truth pixels, especially in high-texture regions, effectively reweighting high-contribution areas.

Take the α-blending formula in Equation (4) as an example. The pose perturbation δT modifies the projected location $P_{j}^{2D}$, which alters the rendering order *m* and, hence, the cumulative opacity product $\prod_{n=1}^{m-1}\left(1-\alpha_{K_{i}}^{n}\right)$. This introduces a nonlinear effect on final pixel values and causes the optimization objective (e.g., PSNR vs. APE) to exhibit non-convex behavior. The proposed LGSBA framework is designed to handle this by jointly optimizing poses and Gaussians to balance localization accuracy with perceptual quality.
